# Supplementary material for: Post-ischemic ubiquitination at the postsynaptic density reversibly influences the activity of ischemia-relevant kinases
Source: Commun Biol. 2024 Mar 13;7:321. doi: 10.1038/s42003-024-06009-8 (PMC10937959; doi:10.1038/s42003-024-06009-8)
Supplement: Supplementary file 2 — Supplementary Information [file 42003_2024_6009_MOESM2_ESM.pdf]

Supplementary Information

accompanying the article "Post-ischemic ubiquitination at the postsynaptic density reversibly influences the activity of ischemia-relevant kinases."

Authors:
Luvna Dhawka[1, #], Victoria Palfini[1, #], Emma Hambright[1], Ismary Blanco[1], Carrie Poon[1], Anja Kahl[1], Ulrike Resch[2], Ruchika Bhawal[3], Corinne Benakis[1, &], Vaishali Balachandran[1], Alana Holder[1], Sheng Zhang[3], Costantino Iadecola[1], Karin Hochrainer[1, *]

[1]Feil Family Brain and Mind Research Institute, Weill Cornell Medicine, New York, New York, USA; [2]Center for Physiology and Pharmacology, Medical University of Vienna, Vienna, Austria; [3]Institute of Biotechnology, Cornell University, Ithaca, New York, USA.

[&]Present address: Institute for Stroke and Dementia Research, Ludwig-Maximilians-University Munich, Munich, Germany.
[#]These authors contributed equally to this work.

*Correspondence: kah2015@med.cornell.edu

This file includes:
Supplementary Tables 1 and 2
Supplementary Figures 1-11

Supplementary Data 1 (proteomics source data), Supplementary Data 2 (functional annotation of proteins with increased post-ischemic ubiquitination) and Supplementary Data 3 (graph and plot source data) are provided as separate .xlsx files.

Supplementary Table 1: **Primary antibodies used for immunoprecipitation.**

Antibody	Clone	Host	IgG subclass	Dilution	Manufacturer	Catalog number
CaMKIIα	CBα-2	Mouse	IgG_{2a}	4μg	Invitrogen	13-7300
CaMKIIβ	CB-β-I	Mouse	IgG_{2b}	4μg	Invitrogen	13-9800
Cdk5	--	Rabbit	--	1:50	Cell Signaling	2506
CKβ	--	Rabbit	--	4μg	Proteintech	15137-1-AP
GluA2	E1L8U	Rabbit	--	1:50	Cell Signaling	13607
GluN1	D65B7	Rabbit	--	1μl/50μg	Cell Signaling	5704
GluN2B	13/NMDAR2B	Mouse	IgG_{2b}	4μg	BD Biosciences	610416
p35/p25	C64B10	Rabbit	--	1:50	Cell Signaling	2680
PKCβ	D3E70	Rabbit	--	1:50	Cell Signaling	46809
PKCγ	D2V6T	Rabbit	--	1:100	Cell Signaling	59090
PSD93	D4Z4D	Rabbit	--	1:50	Cell Signaling	19046
PSD95	6G6-1C9	Mouse	IgG_{2a}	4μg	Invitrogen	MA1-045
Pten	--	Rabbit	--	2μg	Proteintech	22034-1-AP
Pyk2	--	Rabbit	--	4μg	Proteintech	17592-1-AP
Shank2	--	Rabbit	--	1:50	Cell Signaling	12218
Shank3	D5K6R	Rabbit	--	1μl/50μg	Cell Signaling	64555
TrkB	--	Rabbit	--	1:200	Proteintech	13129-1-AP
Isotype control	G3A1	Mouse	IgG_1	4μg	Cell Signaling	5415
Isotype control	MOPC-173	Mouse	IgG_{2a}	4μg	Biolegend	400201
Isotype control	27-35	Mouse	IgG_{2b}	4μg	Biolegend	402201
Isotype control	poly29108	Rabbit	--	4μg	Biolegend	910801

Note: For Cell Signaling antibodies, dilutions are μL antibody per μg total protein

Supplementary Table 2: **Primary antibodies used for Western blotting.**

Antibody	Clone	Host	IgG subclass	Dilution	Manufacturer	Catalog number
β-actin	AC-15	Mouse	IgG_1	1:10000	Sigma	A5441
CaMKIIα	CBα-2	Mouse	IgG_{2a}	1:500	Invitrogen	13-7300
CaMKIIβ	CB-β-I	Mouse	IgG_{2b}	1:1000	Invitrogen	13-9800
Cdk5	--	Rabbit	--	1:1000	Cell Signaling	2506
CKβ	--	Rabbit	--	1:1000	Proteintech	15137-1-AP
Crmp2	C terminus	Rabbit	--	1:1000	ECM Biosciences	CP2161
nNOS	--	Rabbit	--	1:2000	Enzo	BML-SA227
GluA2	E1L8U	Rabbit	--	1:1000	Cell Signaling	13607
GluN1	D65B7	Rabbit	--	1:1000	Cell Signaling	5704
GluN2B	13/NMDAR2B	Mouse	IgG_{2b}	1:500	BD Biosciences	610416
p35/p25	C64B10	Rabbit	--	1:1000	Cell Signaling	2680
PKCβ	D3E70	Rabbit	--	1:1000	Cell Signaling	46809
PKCγ	D2V6T	Rabbit	--	1:1000	Cell Signaling	59090
PSD93	D4Z4D	Rabbit	--	1:1000	Cell Signaling	19046
PSD95	16/PSD-95	Mouse	IgG_1	1:500	BD Biosciences	610495
Pten	--	Rabbit	--	1:2000	Proteintech	22034-1-AP
Pyk2	--	Rabbit	--	1:3000	Proteintech	17592-1-AP
Shank2	--	Rabbit	--	1:1000	Cell Signaling	12218
Shank3	D5K6R	Rabbit	--	1:1000	Cell Signaling	64555
Src	36D10	Rabbit	--	1:1000	Cell Signaling	2109
Tau	TAU-5	Mouse	IgG_1	1:200	Invitrogen	MA5-12808
TrkB	--	Rabbit	--	1:1000	Proteintech	13129-1-AP
Ubiquitin	Ubi-1	Mouse	IgG_1	1:500	Invitrogen	13-1600
Ubiquitin K48	Apu2	Rabbit	--	1:1000	Millipore	05-1307
Ubiquitin K63	D7A11	Rabbit	--	1:1000	Cell Signaling	5621
Phospho-Crmp2 (S522)	--	Rabbit	--	1:1000	ECM Biosciences	CP2191
Phospho-GluN2B (Y1472)	--	Rabbit	--	1:1000	Cayman Chemical	10009761
Phospho-GluN2B (S1303)	--	Rabbit	--	1µg/mL	Millipore	07-398
Phospho-nNOS (S847)	--	Rabbit	--	1µg/mL	Abcam	ab16650
Phospho-Pyk2 (Y402)	--	Rabbit	--	1:1000	Invitrogen	44-618G
Phospho-serine/threonine	--	Rabbit	--	1:1000	Cell Signaling	9631
Phospho-Src (Y419)	D49G4	Rabbit	--	1:1000	Cell Signaling	6943
Phospho-Tau (Y18)	9G3	Mouse	IgG_{2a}	1:1000	Novus Biologicals	NBP2-42402
Phospho-Tau (S202/205)	AT8	Mouse	IgG_1	1:500	Invitrogen	MN1020
Phospho-tyrosine	P-Tyr-100	Mouse	IgG_1	1:2000	Cell Signaling	9411

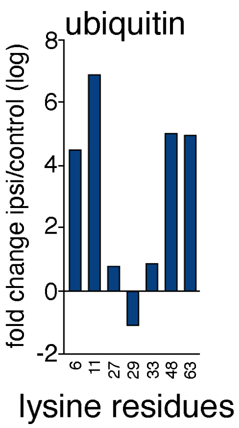

Supplementary Fig. 1. **Fold change in ubiquitin lysine-chain composition after MCAO.** Ubiquitinated lysine residues (K6, K11, K27, K29, K33, K48, and K63) in ubiquitin as identified by nanoLC-MS/MS. Mean fold change ipsilateral vs control (pooled sham and contralateral) cortex is shown on a $\log_{10}$ scale (n=2-3 MS runs; pooled 20 mice/group for each run).

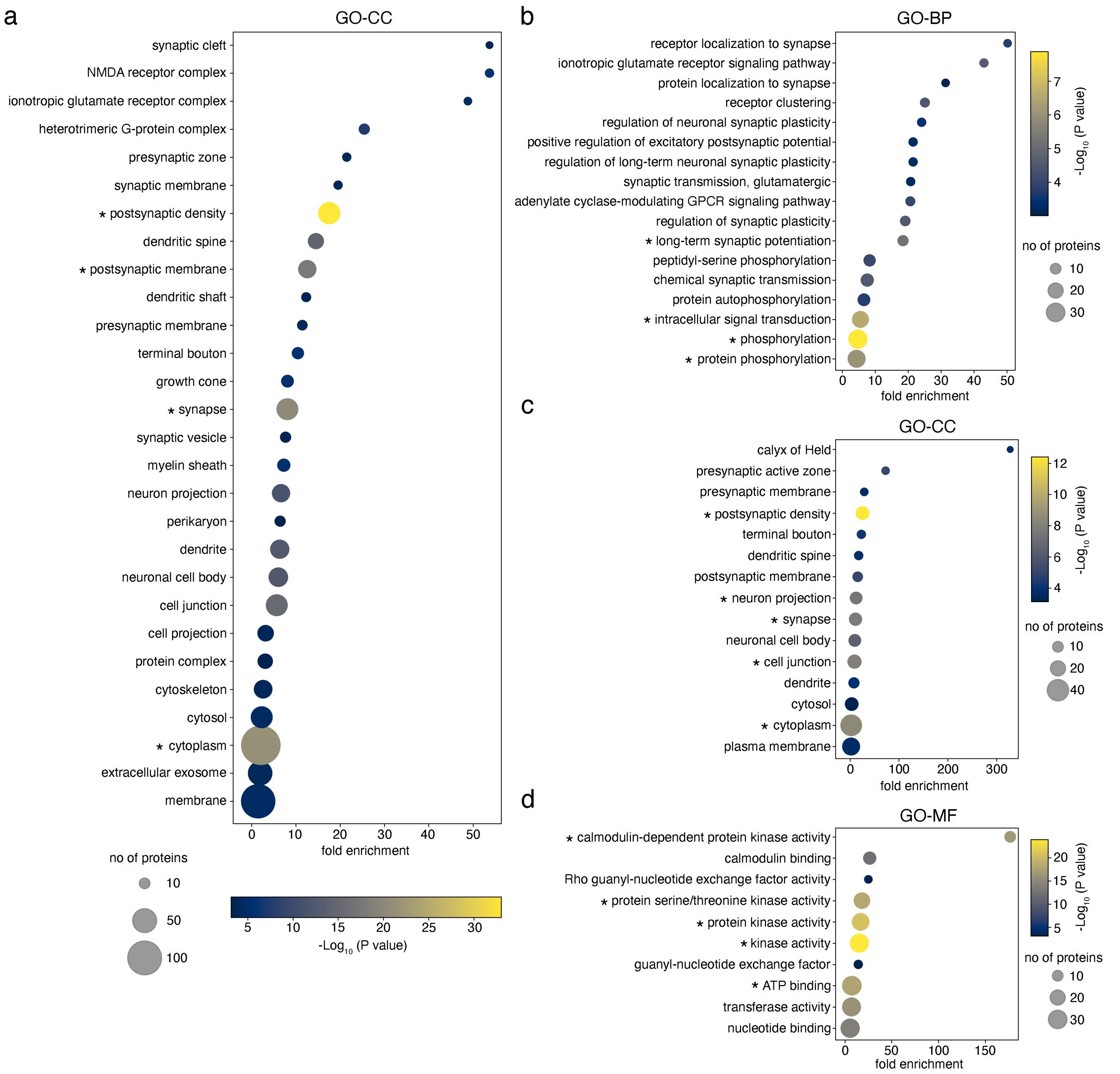

Supplementary Fig. 2. **Dotplots showing GO enrichment analyses of proteins with increased post-ischemic ubiquitination. (a)** GO analysis for cellular compartment accompanying Figure 3a. **(b)** GO analysis for biological process accompanying Figure 4a. **(c)** GO analysis for cellular compartment accompanying Figure 4b. **(d)** GO analysis for molecular function accompanying Figure 4b. Asterisks in each plot mark the 4-5 terms with the most significant enrichment. Only terms with a P value <0.001 are shown.

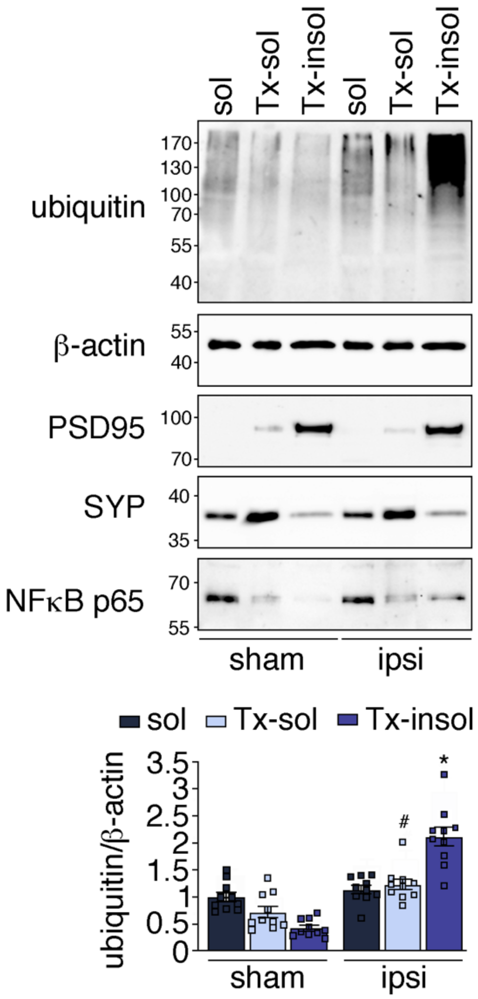

Supplementary Fig. 3. **The ubiquitin-containing Tx-insoluble fraction stains positive for the postsynaptic density marker PSD95.** Ubiquitin was detected in cortical soluble (sol), Tx-soluble (Tx-sol) and Tx-insoluble (Tx-insol) fractions derived from mice that underwent sham or MCAO/1h reperfusion (ipsi) surgeries. The Tx-insol fraction was also stained with PSD95, showing that this fraction contains the postsynaptic density. Markers for cytosol (p65) and synaptic membranes (SYP) were found in sol and Tx-sol fractions, respectively. Results were quantified. $^{\#}P=0.0023$ from Tx-sol sham; $^{*}P<0.0001$ from Tx-insol sham; two-tailed unpaired t-test; n=10 mice/group. Data are expressed as mean ± s.e.m.

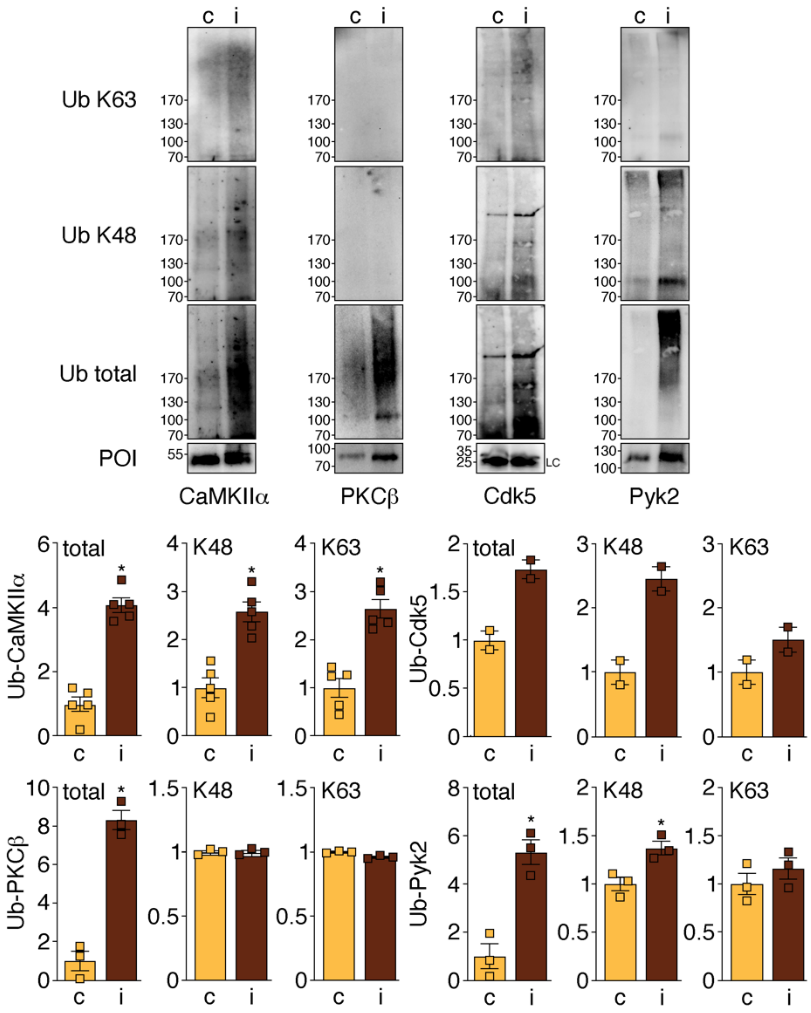

Supplementary Fig. 4. **Kinases are modified by different ubiquitin chain types after ischemia.** Kinases were immunoprecipitated from cortical ipsilateral and contralateral PSD lysates after MCAO and 1hr reperfusion, and presence of K63- and K48-ubiquitin was assessed by Western Blotting with respective antibodies. Staining for total ubiquitin served as control for successful co-IP of ubiquitin. Results were quantified. CaMKIIα: total Ub *P<0.0001, K48 Ub *P=0.0006, K63 Ub *P=0.0003; PKCβ: total Ub *P=0.0005; Pyk2: total Ub *P=0.0041, K48 Ub *P=0.0199; two-tailed unpaired t-test; n=3-5 animals/group. Due to low replicate number, Cdk5 data were not statistically evaluated. Data are expressed as mean ± s.e.m. c, contralateral; i, ipsilateral; LC, light-chain; Ub, ubiquitin.

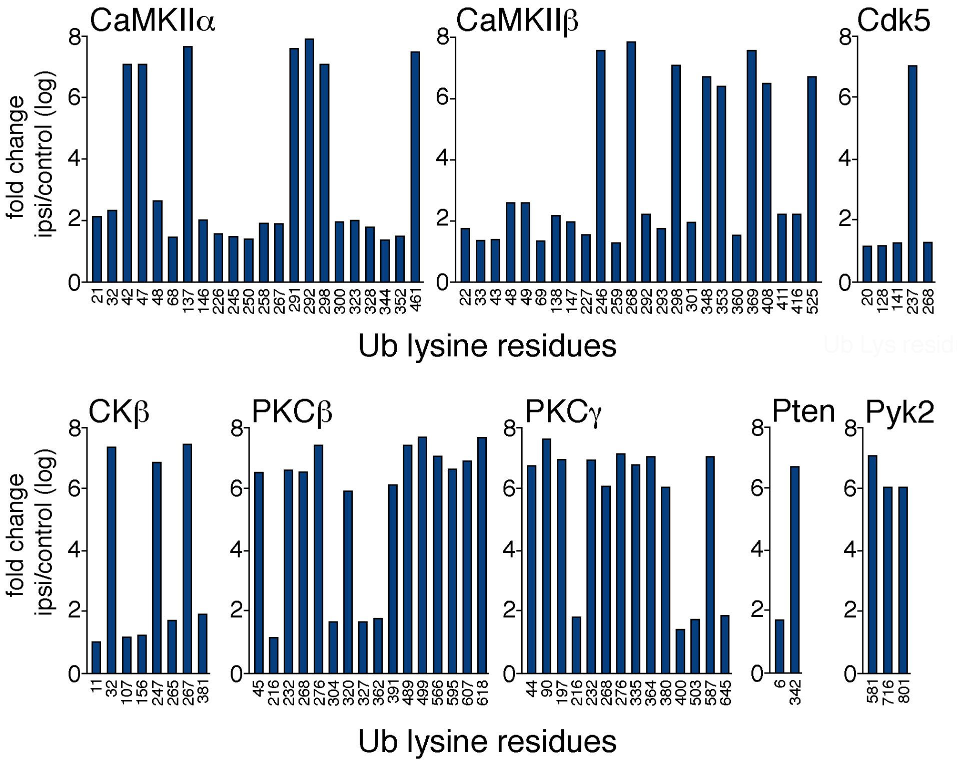

Supplementary Fig. 5. **Fold increase of ubiquitination across lysine residues in PSD-associated kinases and phosphatases after MCAO.** Ubiquitinated lysine residues in the kinases CaMKIIα, CaMKIIβ, PKCβ, PKCγ, Cdk5, CKβ, and Pyk2, and the phosphatase Pten as identified by nanoLC-MS/MS. Mean fold change ipsilateral vs control (pooled sham and contralateral) cortex is shown on a $\log_{10}$ scale (n=2-3 MS runs; pooled 20 mice/group for each run). Ub, ubiquitin.

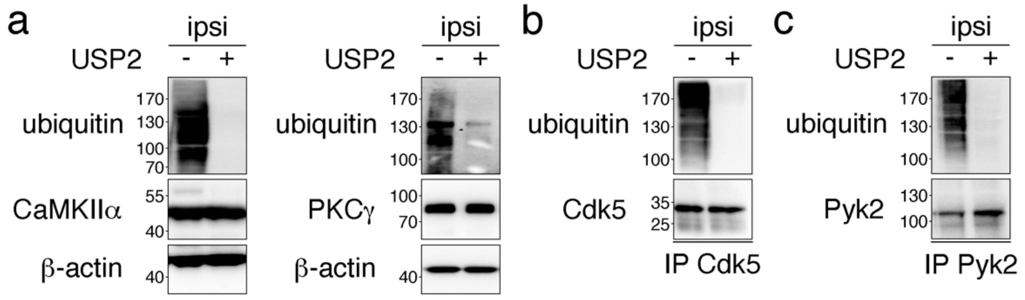

Supplementary Fig. 6. **USP2 efficiently removes ubiquitin from substrates. (a)** Total PSD lysates or **(b) (c)** precipitated PSD-kinases were incubated with recombinant USP2, and high molecular weight ubiquitin smear as well as respective kinases were detected by Western Blot. For lysates, β-actin was used as loading control. ipsi, ipsilateral.

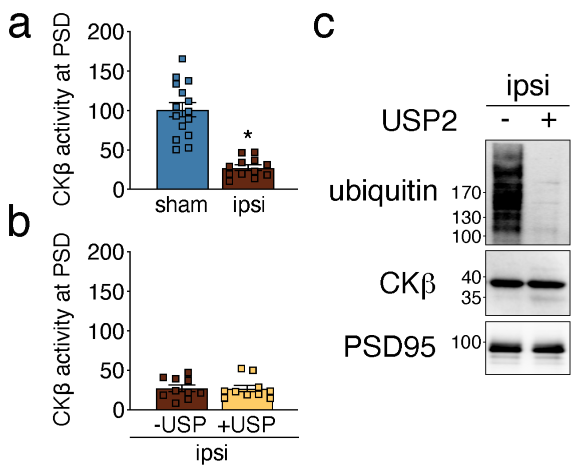

Supplementary Fig. 7. **The effect of MCAO/reperfusion and USP2 treatment on CKβ activity. (a)** CKβ activity was determined in cortical PSD lysates from sham and stroked mice. *P<0.0001; two-tailed unpaired t-test; n=12-15 animals/group. Data are expressed as mean ± s.e.m. **(b)** PSD lysates from stroked mice were treated with USP2 and CKβ activity was reassessed. n=10 animals. **(c)** USP2 digest was verified by Western Blotting as in described in Supplementary Fig. 6. CKβ and PSD95 were loaded as controls. ipsi, ipsilateral.

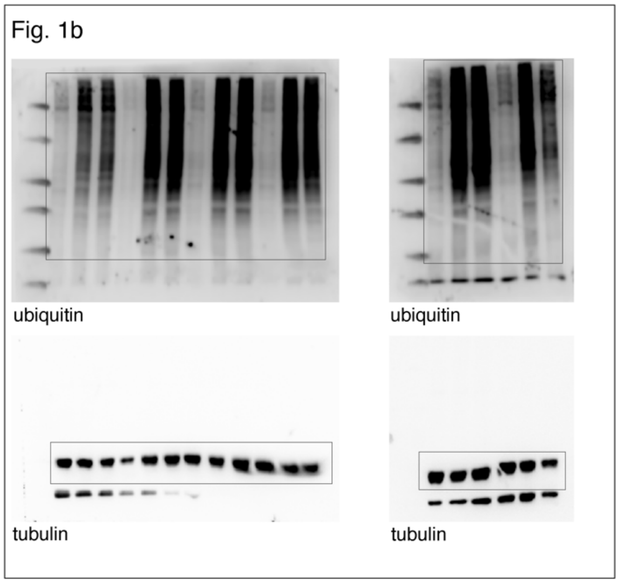

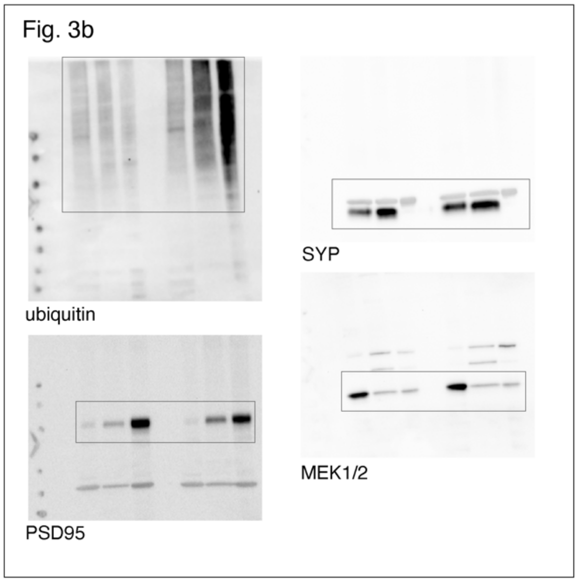

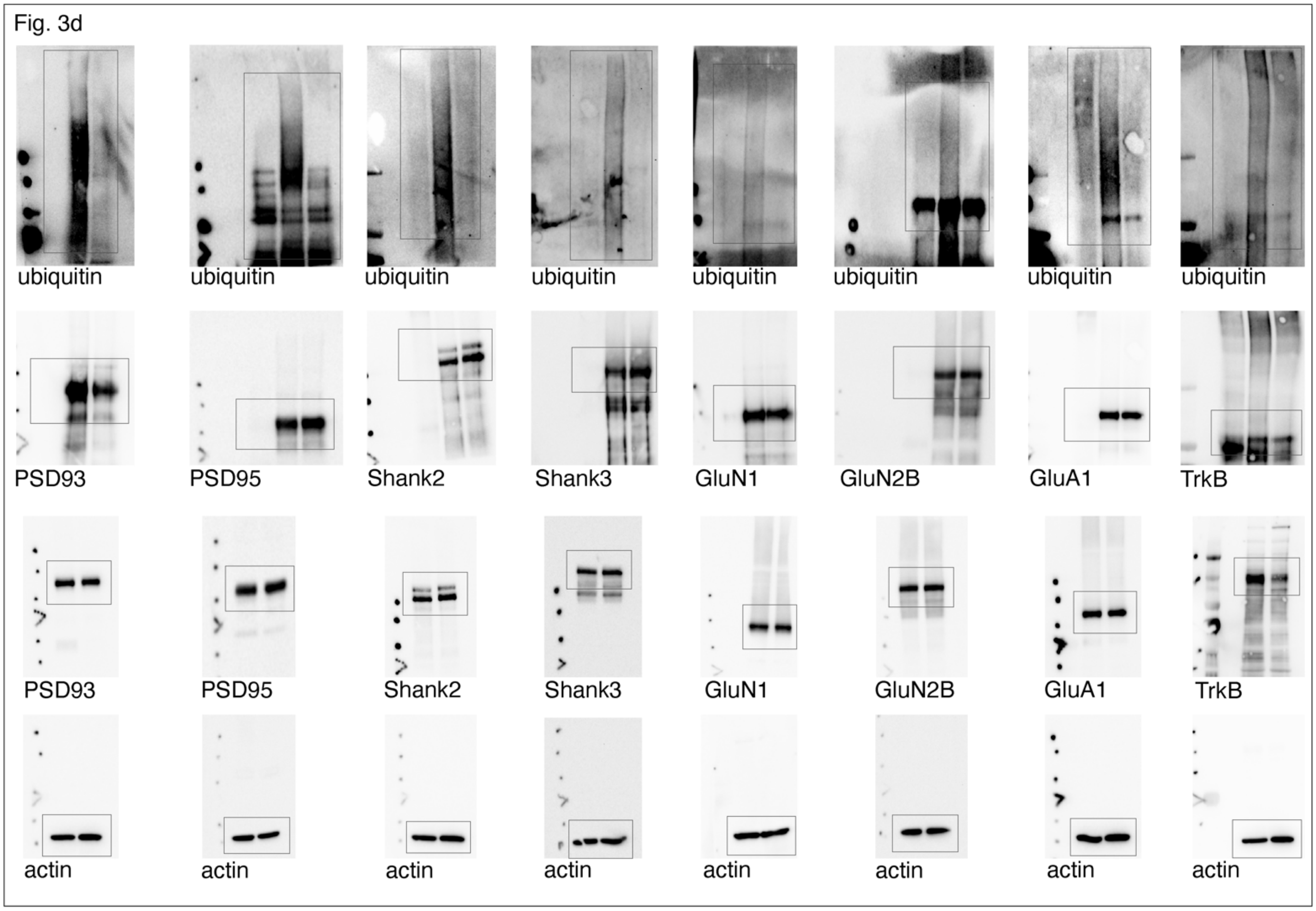

Supplementary Fig. 8. **Uncropped and unedited Western Blot images underlying Figs. 1b, 3b, and 3d.**

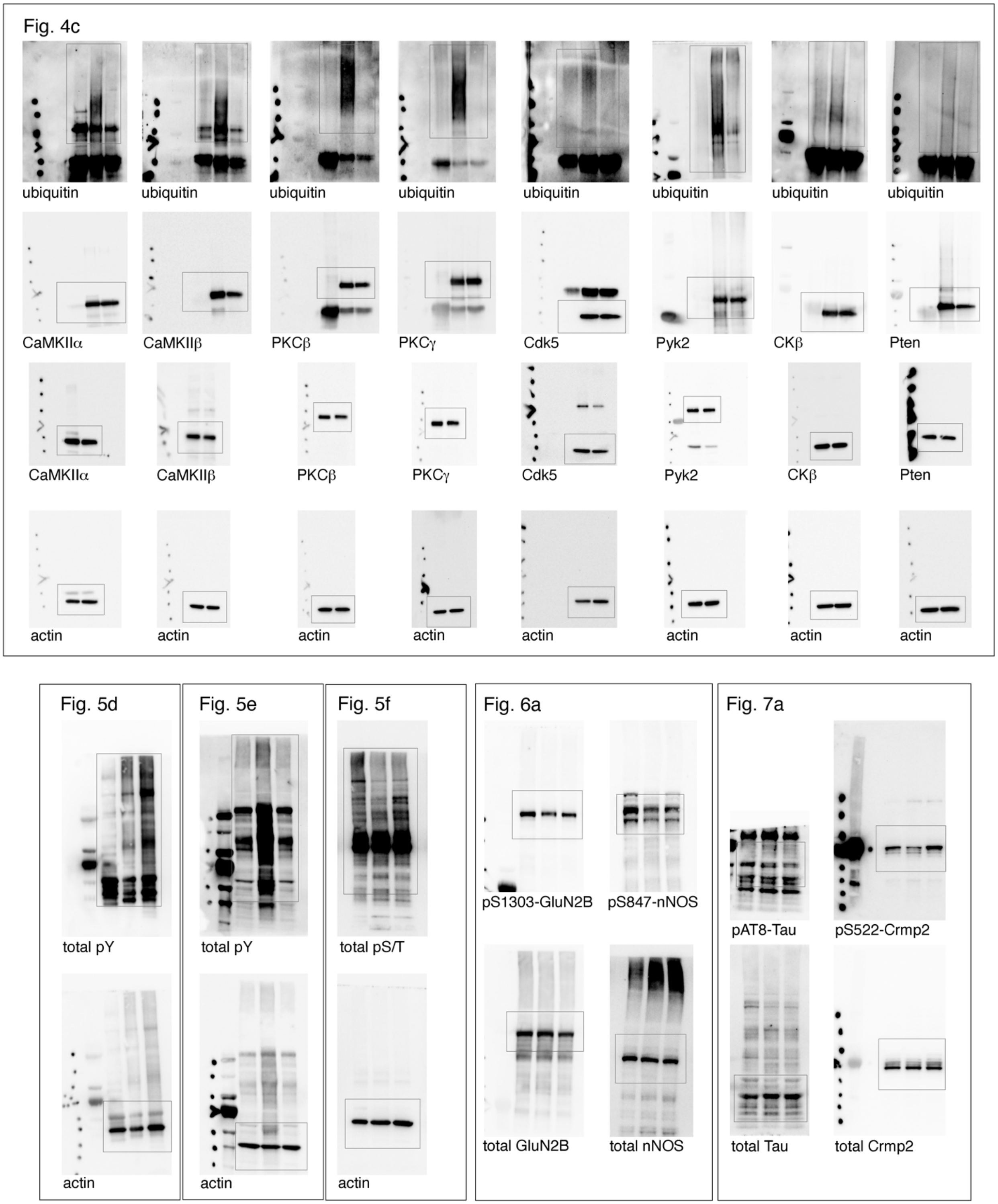

Supplementary Fig. 9. **Uncropped and unedited Western Blot images underlying Figs. 4c, 5d, 5e, 5f, 6a, and 7a.**

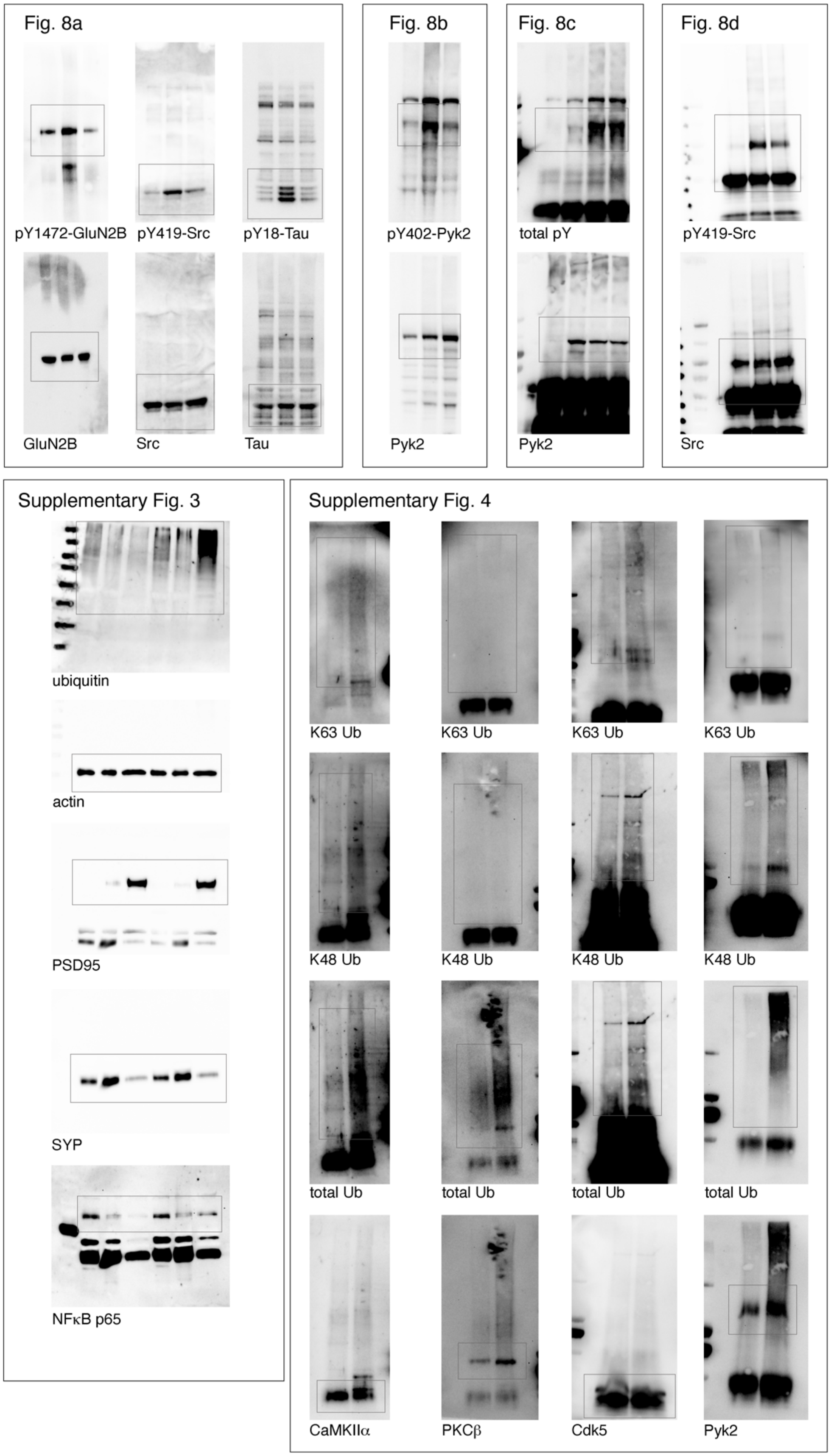

Supplementary Fig. 10. **Uncropped and unedited Western Blot images underlying Figs. 8a, 8b, 8c, 8d, as well as Supplementary Figs. 3 and 4.**

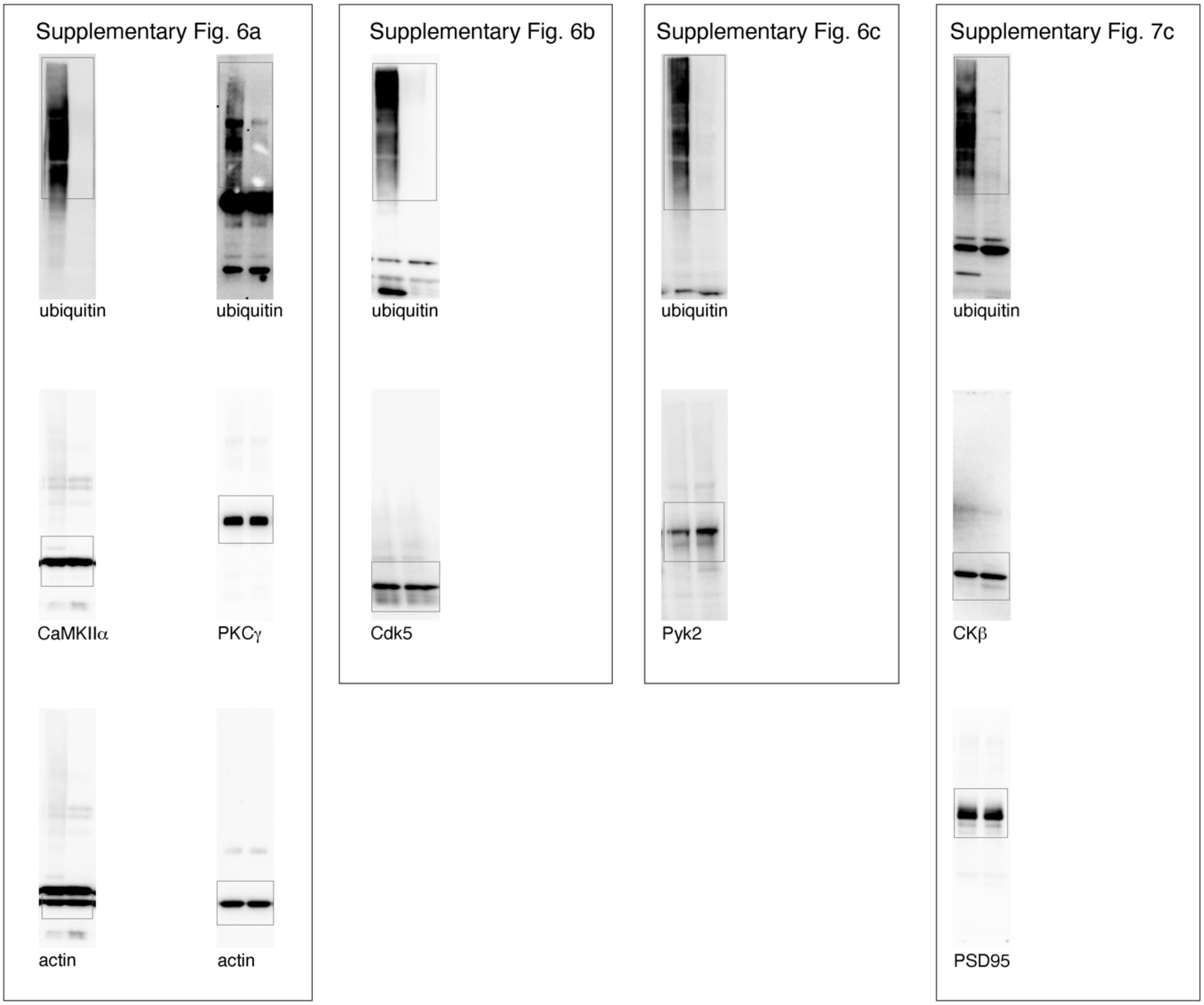

Supplementary Fig. 11. **Uncropped and unedited Western Blot images underlying Supplementary Figs. 6a, 6b, 6c, and 7c.**
