# Supplementary material for: Post-ischemic ubiquitination at the postsynaptic density reversibly influences the activity of ischemia-relevant kinases
Source: Commun Biol. 2024 Mar 13;7:321. doi: 10.1038/s42003-024-06009-8 (PMC10937959; doi:10.1038/s42003-024-06009-8)
Supplement: Supplementary file 3 — Description of additional supplementary files [file 42003_2024_6009_MOESM3_ESM.pdf]

Description of Additional Supplementary Files

File name: Supplementary Data 1

Description: Log-transformed proteomics source data.

File name: Supplementary Data 2

Description: Functional annotation of proteins with increased post-ischemic ubiquitination.

File name: Supplementary Data 3

Description: Graph and plot source data.
