# Supplementary material for: Post-ischemic ubiquitination at the postsynaptic density reversibly influences the activity of ischemia-relevant kinases
Source: Commun Biol. 2024 Mar 13;7:321. doi: 10.1038/s42003-024-06009-8 (PMC10937959; doi:10.1038/s42003-024-06009-8)
Supplement: Supplementary file 7 — Reporting Summary [file 42003_2024_6009_MOESM7_ESM.pdf]

nature portfolio

Corresponding author(s): Karin Hochrainer

Last updated by author(s): Feb 6, 2024

Reporting Summary

Nature Portfolio wishes to improve the reproducibility of the work that we publish. This form provides structure for consistency and transparency in reporting. For further information on Nature Portfolio policies, see our Editorial Policies and the Editorial Policy Checklist.

Please do not complete any field with "not applicable" or n/a. Refer to the help text for what text to use if an item is not relevant to your study.
For final submission: please carefully check your responses for accuracy; you will not be able to make changes later.

Statistics

For all statistical analyses, confirm that the following items are present in the figure legend, table legend, main text, or Methods section.

n/a	Confirmed	
☐	☒	The exact sample size (n) for each experimental group/condition, given as a discrete number and unit of measurement
☐	☒	A statement on whether measurements were taken from distinct samples or whether the same sample was measured repeatedly
☐	☒	The statistical test(s) used AND whether they are one- or two-sided<br>*Only common tests should be described solely by name; describe more complex techniques in the Methods section.*
☒	☐	A description of all covariates tested
☒	☐	A description of any assumptions or corrections, such as tests of normality and adjustment for multiple comparisons
☐	☒	A full description of the statistical parameters including central tendency (e.g. means) or other basic estimates (e.g. regression coefficient) AND variation (e.g. standard deviation) or associated estimates of uncertainty (e.g. confidence intervals)
☐	☒	For null hypothesis testing, the test statistic (e.g. F, t, r) with confidence intervals, effect sizes, degrees of freedom and P value noted<br>*Give P values as exact values whenever suitable.*
☒	☐	For Bayesian analysis, information on the choice of priors and Markov chain Monte Carlo settings
☒	☐	For hierarchical and complex designs, identification of the appropriate level for tests and full reporting of outcomes
☒	☐	Estimates of effect sizes (e.g. Cohen's d, Pearson's r), indicating how they were calculated

Our web collection on statistics for biologists contains articles on many of the points above.

Software and code

Policy information about availability of computer code

Data collection	Proteome: The nanoLC-MS/MS data acquisition was carried out using an Orbitrap Fusion mass spectrometer (Thermo Fisher Scientific) equipped with a nano spray Flex Ion Source with high energy collision dissociation (HCD) and coupled with UltiMate3000 RSLCnano (Dionex, Sunnyvale, CA). Data were acquired under Xcalibur 3.0 operation software and Orbitrap Fusion Tune 2.0 (Thermo Fisher Scientific). Western Blotting: Proteins were visualized on a ChemiDoc imager (Bio-Rad Laboratories). Radioactive kinase activity measurement: Radioactivity was quantified in a LS3801 scintillation counter (Beckman Coulter) by the Cherenkov method.
Data analysis	Prior to MS analysis, protein bands were visualized with colloidal Coomassie blue stain (Thermo Fisher Scientific) and quantified with a Typhoon 9400 scanner by ImageQuant Software version TL 8.1 (GE Healthcare, Chicago, IL). Global, phospho- and ubiquitin-enriched MS-raw data were analyzed by MaxQuant version 1.6.0.1 (Max Planck Institute of Biochemistry, Martinsried, Germany) and Proteome Discoverer-Sequest (PD) 2.2. algorithms against the UniProt mouse database (UP000000589_10090, download 2018), respectively. Abundance binning was performed with the Perseus software (https://maxquant.net/perseus/). The single-cell sequencing database Dropviz (http://dropviz.org/) was employed to determine the brain cell type with the highest expression of ubiquitinated proteins. Protein interaction network analysis was performed using interaction data (text mining and experiments) from the Search Tool for the Retrieval of Interacting Genes/proteins (STRING) database (https://string-db.org/). Gene Ontology (GO) enrichment analysis was performed using the functional annotation tool of the DAVID bioinformatics resources (Version 6.8) (https://david.ncifcrf.gov/). Proteomics results were visualized with Python-based software platforms Instant Clue (Version 0.10.10, University of Cologne, Germany) and Orange3 (Version 3.35.0, University of Ljubljana, Slovenia), as well as GraphPad Prism (Version 9.5.1). Li-Cor Image Studio (v5.2.5) and Biorad Image Lab (v6.1) were used for quantification analysis of Western Blots. GraphPad Prism (Version 9.5.1) software was used for statistical analyses.

For manuscripts utilizing custom algorithms or software that are central to the research but not yet described in published literature, software must be made available to editors and reviewers. We strongly encourage code deposition in a community repository (e.g. GitHub). See the Nature Portfolio guidelines for submitting code & software for further information.

Data

Policy information about availability of data

All manuscripts must include a data availability statement. This statement should provide the following information, where applicable:

- Accession codes, unique identifiers, or web links for publicly available datasets
- A description of any restrictions on data availability
- For clinical datasets or third party data, please ensure that the statement adheres to our policy

Proteomics data have been deposited to the ProteomeXchange Consortium (http://proteomecentral.proteomexchange.org) via the PRIDE partner repository with the dataset identifier PXD042951. Proteomics ubiquitination data can also be interrogated via https://hochrainerlab.shinyapps.io/StrokeUbiOmics/. Extracted proteomics source data converted to log-scale changes can be found in Table S1, tabs 1-4. Other source data underlying analyses for plots and graphs are available from the corresponding author upon request.

Research involving human participants, their data, or biological material

Policy information about studies with human participants or human data. See also policy information about sex, gender (identity/presentation), and sexual orientation and race, ethnicity and racism.

Reporting on sex and gender	N/A
Reporting on race, ethnicity, or other socially relevant groupings	N/A
Population characteristics	N/A
Recruitment	N/A
Ethics oversight	N/A

Note that full information on the approval of the study protocol must also be provided in the manuscript.

Field-specific reporting

Please select the one below that is the best fit for your research. If you are not sure, read the appropriate sections before making your selection.

☒ Life sciences ☐ Behavioural & social sciences ☐ Ecological, evolutionary & environmental sciences

Life sciences study design

All studies must disclose on these points even when the disclosure is negative.

Sample size	Sample size for proteomics studies was calculated according to published work citing how much mg protein is needed for successful detection of ubiquitin moieties by mass spectrometry. Sample size for biochemical analyses was determined according to power analysis based on previous published works by our lab. Semi-quantitative methods, such as PSD isolation, immunoprecipitation (IP), Western Blotting (WB), etc. were carried out 3-7 times to ensure reproducibility.
Data exclusions	Low abundance proteins due to the natural limitation in mass spectrometry are at detection limit and therefore detected with high variability. To account for this, we performed outlier analysis for determining changes in ubiquitination levels of proteins (Figure 2A) by the interquartile range (IQR) method (+/- 1.5x IQR). We detected 135 outliers out of 1845 hits in sham and contralateral control samples, and 0 outliers out of 1107 hits in ipsilateral samples. Further, we performed outlier analysis with the same method for Pyk2 Y402 phosphorylation (Figure 8B), which was variably elevated across different reperfusion times. Here, we excluded 2 outliers out of 6 replicates for the 30min and 1 outlier out of 6 replicates for the 60min time-points.
Replication	At least three independent experiments were performed to support each finding in the manuscript. All attempts at replication were successful and are included in the figures.
Randomization	Sham and MCAO surgeries were performed in randomly assigned mice.
Blinding	Data analyses were performed blind whenever possible.

Reporting for specific materials, systems and methods

We require information from authors about some types of materials, experimental systems and methods used in many studies. Here, indicate whether each material, system or method listed is relevant to your study. If you are not sure if a list item applies to your research, read the appropriate section before selecting a response.

Materials & experimental systems

n/a	Involved in the study	
☐	☒	Antibodies
☒	☐	Eukaryotic cell lines
☒	☐	Palaeontology and archaeology
☐	☒	Animals and other organisms
☒	☐	Clinical data
☒	☐	Dual use research of concern
☒	☐	Plants

Methods

n/a	Involved in the study	
☒	☐	ChIP-seq
☒	☐	Flow cytometry
☒	☐	MRI-based neuroimaging

Antibodies

Antibodies used

Primary antibodies used for immunoprecipitation:
Antibody / Clone / Host / IgG subclass / Dilution / Manufacturer / Catalog number
CaMKIIa CBa-2 Mouse IgG2a 4µg Invitrogen 13-7300
CaMKIIb CB-b-I Mouse IgG2b 4µg Invitrogen 13-9800
Cdk5 -- Rabbit -- 1:50 Cell Signaling 2506
CKb -- Rabbit -- 4µg Proteintech 15137-1-AP
GluA2 E1L8U Rabbit -- 1:50 Cell Signaling 13607
GluN1 D65B7 Rabbit -- 1µl/50µg Cell Signaling 5704
GluN2B 13/NMDAR2B Mouse IgG2b 4µg BD Biosciences 610416
p35/p25 C64B10 Rabbit -- 1:50 Cell Signaling 2680
PKCb D3E70 Rabbit -- 1:50 Cell Signaling 46809
PKCg D2V6T Rabbit -- 1:100 Cell Signaling 59090
PSD93 D4Z4D Rabbit -- 1:50 Cell Signaling 19046
PSD95 6G6-1C9 Mouse IgG2a 4µg Invitrogen MA1-045
Pten -- Rabbit -- 2µg Proteintech 22034-1-AP
Pyk2 -- Rabbit -- 4µg Proteintech 17592-1-AP
Shank2 -- Rabbit -- 1:50 Cell Signaling 12218
Shank3 D5K6R Rabbit -- 1µl/50µg Cell Signaling 64555
TrkB -- Rabbit -- 1:200 Proteintech 13129-1-AP
Isotype control G3A1 Mouse IgG1 4µg Cell Signaling 5415
Isotype control MOPC-173 Mouse IgG2a 4µg Biolegend 400201
Isotype control 27-35 Mouse IgG2b 4µg Biolegend 402201
Isotype control poly29108 Rabbit -- 4µg Biolegend 910801
Note: For Cell Signaling antibodies, dilutions are µL antibody per µg total protein

Primary antibodies used for Western blotting:
Antibody / Clone / Host / IgG subclass / Dilution / Manufacturer / Catalog number
b-actin AC-15 Mouse IgG1 1:10000 Sigma A5441
CaMKIIa CBa-2 Mouse IgG2a 1:500 Invitrogen 13-7300
CaMKIIb CB-b-I Mouse IgG2b 1:1000 Invitrogen 13-9800
Cdk5 -- Rabbit -- 1:1000 Cell Signaling 2506
CKb -- Rabbit -- 1:1000 Proteintech 15137-1-AP
Crmp2 C terminus Rabbit -- 1:1000 ECM Biosciences CP2161
nNOS -- Rabbit -- 1:2000 Enzo BML-SA227
GluA2 E1L8U Rabbit -- 1:1000 Cell Signaling 13607
GluN1 D65B7 Rabbit -- 1:1000 Cell Signaling 5704
GluN2B 13/NMDAR2B Mouse IgG2b 1:500 BD Biosciences 610416
p35/p25 C64B10 Rabbit -- 1:1000 Cell Signaling 2680
PKCb D3E70 Rabbit -- 1:1000 Cell Signaling 46809
PKCg D2V6T Rabbit -- 1:1000 Cell Signaling 59090
PSD93 D4Z4D Rabbit -- 1:1000 Cell Signaling 19046
PSD95 16/PSD-95 Mouse IgG1 1:500 BD Biosciences 610495
Pten -- Rabbit -- 1:2000 Proteintech 22034-1-AP
Pyk2 -- Rabbit -- 1:3000 Proteintech 17592-1-AP
Shank2 -- Rabbit -- 1:1000 Cell Signaling 12218
Shank3 D5K6R Rabbit -- 1:1000 Cell Signaling 64555
Src 36D10 Rabbit -- 1:1000 Cell Signaling 2109
Tau TAU-5 Mouse IgG1 1:200 Invitrogen MA5-12808
TrkB -- Rabbit -- 1:1000 Proteintech 13129-1-AP
Ubiquitin Ubi-1 Mouse IgG1 1:500 Invitrogen 13-1600
Ubiquitin K48 Apu2 Rabbit -- 1:1000 Millipore 05-1307
Ubiquitin K63 D7A11 Rabbit -- 1:1000 Cell Signaling 5621
Phospho-Crmp2 (S522) -- Rabbit -- 1:1000 ECM Biosciences CP2191
Phospho-GluN2B (Y1472) -- Rabbit -- 1:1000 Cayman Chemical 10009761
Phospho-GluN2B (S1303) -- Rabbit -- 1µg/mL Millipore 07-398
Phospho-nNOS (S847) -- Rabbit -- 1µg/mL Abcam ab16650
Phospho-Pyk2 (Y402) -- Rabbit -- 1:1000 Invitrogen 44-618G

	Phospho-serine/threonine -- Rabbit -- 1:1000 Cell Signaling 9631<br>Phospho-Src (Y419) D49G4 Rabbit -- 1:1000 Cell Signaling 6943<br>Phospho-Tau (Y18) 9G3 Mouse IgG2a 1:1000 Novus Biologicals NBP2-42402<br>Phospho-Tau (S202/205) AT8 Mouse IgG1 1:500 Invitrogen MN1020<br>Phospho-tyrosine P-Tyr-100 Mouse IgG1 1:2000 Cell Signaling 9411
Validation	All used antibodies were extensively validated by us, the manufacturer and/or in the literature with the following methods: for western blotting a) tagged over-expression controls, b) knockdown or, if available, knockout controls, c) blocking antibody controls; and for immunoprecipitation a) isotype control precipitations, b) blank agarose/sepharose controls, c) blocking antibody controls. Phospho-specific antibodies were assessed for specificity by comparing alkaline phosphatase-treated with non-treated samples.

Animals and other research organisms

Policy information about studies involving animals; ARRIVE guidelines recommended for reporting animal research, and Sex and Gender in Research

Laboratory animals	All animal procedures were approved by the Weill Cornell Medicine Institutional Animal Care and Use Committee (IACUC) and were carried out according to IACUC, NIH, and ARRIVE guidelines (http://www.nc3rs.org/ARRIVE). Transient focal cerebral ischemia was induced in 8-12 weeks old C57BL6/J WT male mice (Jackson Laboratories, Bar Harbor, ME) via middle cerebral artery occlusion (MCAO) using an intraluminal filament.
Wild animals	Wilds animals were not investigated in the study.
Reporting on sex	All experiments were performed in 8-12 weeks old male mice.
Field-collected samples	Field-collected samples were not studied in the study.
Ethics oversight	All procedures were approved by the Institutional Animal Care and Use Committee of Weill Cornell Medicine.

Note that full information on the approval of the study protocol must also be provided in the manuscript.

This checklist template is licensed under a Creative Commons Attribution 4.0 International License, which permits use, sharing, adaptation, distribution and reproduction in any medium or format, as long as you give appropriate credit to the original author(s) and the source, provide a link to the Creative Commons license, and indicate if changes were made. The images or other third party material in this article are included in the article's Creative Commons license, unless indicated otherwise in a credit line to the material. If material is not included in the article's Creative Commons license and your intended use is not permitted by statutory regulation or exceeds the permitted use, you will need to obtain permission directly from the copyright holder. To view a copy of this license, visit http://creativecommons.org/licenses/by/4.0/
